# Thyroid Cancer Presenting as Neck Pain at a Chiropractic Clinic

**DOI:** 10.7759/cureus.39276

**Published:** 2023-05-20

**Authors:** Benjamin Cheong, Hans Juin Ho Teh, Gabriel Siu Nam Ng, Kevin Hsu Kai Huang

**Affiliations:** 1 Chiropractic and Physiotherapy Centre, New York Medical Group, Hong Kong, HKG; 2 Chiropractic and Physiotherapy Centre, New York Medical Group, Hong Kong, CHN

**Keywords:** neck pain, spinal metastasis, chiropractor, chiropractic, thyroid cancer

## Abstract

We report an unusual case of metastatic papillary thyroid carcinoma presenting with progressive neck pain in a 58-year-old female with known bilateral thyroid nodules. Despite initial benign ultrasonographic findings and trials of conservative therapy for over 2 months, the patient’s anterior neck pain and unremitting symptoms prompted concern regarding an underlying orthopedic condition. She sought chiropractic consultation, and MRI revealed pathologic vertebral fractures. Biopsy of the thyroid and vertebral bone lesions demonstrated metastatic thyroid carcinoma. The patient was diagnosed with papillary thyroid cancer. The early detection of metastatic disease is critical for optimizing oncological treatment and outcomes. This case highlights the importance of close follow-up when the initial workup or management fails, consideration of sinister pathologies in high-risk patients, and a multidisciplinary approach to complex conditions. It provides an important reminder not to attribute unresolved symptoms to benign causes without serial re-evaluation, especially in patients with known risks, such as thyroid disease. A high index of suspicion and openness to atypical disease presentations is fundamental to avoid missed opportunities for diagnosis and prompt treatment.

## Introduction

Thyroid cancer is an uncommon malignancy, accounting for approximately 1% of all new malignant diseases, approximately 1.5% in women and 0.5% in men [1]. The metastatic spread of thyroid carcinoma typically occurs in the regional lymph nodes of the neck and mediastinum. Distant metastases are observed in less than 15% of patients at the time of diagnosis, commonly in the bones and lungs [1]. Although relatively rare, its incidence has been increasing steadily for decades [1]. Much of the rise in incidence can be attributed to improved diagnostic methods such as high-resolution ultrasonography allowing the detection of small subclinical thyroid nodules [1].

Metastatic thyroid cancer presenting with neck pain is an unusual initial manifestation that can lead to a delayed or missed diagnosis if the primary malignancy is occult or has not been fully evaluated. Chiropractors undergo training and education to develop expertise in spinal pathology and neurology [2-3]. Although serious pathological conditions are infrequently encountered in chiropractic clinics [4], chiropractors are skilled in effectively managing pain with minimal side effects [5]. Chiropractic care is commonly used in determining the cause of neck pain and providing preventative and nonpharmacological treatment options [6]. In this case report, we aim to raise clinical awareness of metastatic thyroid cancer as a rare differential diagnosis for severe or worsening neck pain, particularly when associated risk factors such as thyroid disease are present. This case highlights the importance of a high index of suspicion, close follow-up, and a multidisciplinary approach in patients with unresolved neck pain and known risks, such as thyroid disease. Timely diagnosis and appropriate management of metastatic thyroid cancer can significantly impact patient outcomes and quality of life.

## Case presentation

A 58-year-old female presented to a chiropractor in Hong Kong with a 2-month history of progressive and severe cervicothoracic pain localized to the left side, radiating anteriorly to the left neck and upper trapezius. She described the pain as constant and dull, and rated it 6-7 in severity on a 10-point scale. The pain began after sustaining a distal radius fracture of the left hand following a mechanical fall. Open-reduction internal fixation was not recommended. The fracture exacerbated neck pain and restricted cervical spine movements, which remained unchanged in any position, and was partially alleviated for up to 2 h with hydrocodone/acetaminophen and massage therapy. Initially, the patient consulted a general practitioner who ordered cervical ultrasonography showing bilateral thyroid nodules, possibly adenomatous and reactive lymphadenopathy, without any acute abnormality correlating with the symptoms. She subsequently consulted a traditional Chinese medical practitioner, who provided temporary analgesia with a massage.

The patient denied any changes in bowel or bladder function, nausea or emesis, weight loss, nocturnal pain, numbness, or upper limb weakness. Past medical history included well-managed stage 1 hypertension and hypercholesterolemia. There is no reported family history of thyroid disease, neoplasia or musculoskeletal diseases, as there are no known cases of thyroid cancer, thyroid nodules, or hypothyroidism/hyperthyroidism in the patient's first-degree relatives. Social history was unremarkable. The patient was a lifelong nonsmoker who denied the use of recreational drugs or excess alcohol. Current medications included atorvastatin, lisinopril, aspirin 81 mg daily, and hydrocodone/acetaminophen 10/325 mg every 6 h for pain.

Vital signs were within normal limits. Physical examination revealed a well-nourished female with a guarded cervical spine and limited cervical range of motion (flexion, 50°/80°; extension, 10°/70°; and bilateral rotation, 40°/90°). Muscle palpation elicited hypertonicity and tenderness over the left sternocleidomastoid, splenius capitis, and upper trapezius, without step-offs or masses. The Spurling’s test was positive on the left side, resulting in pain and paresthesia radiating to the occiput. The reflexes were +2 and were equal bilaterally. No deficits were noted in motor or sensory testing of the C5-T1 distribution.

**Figure 1:**
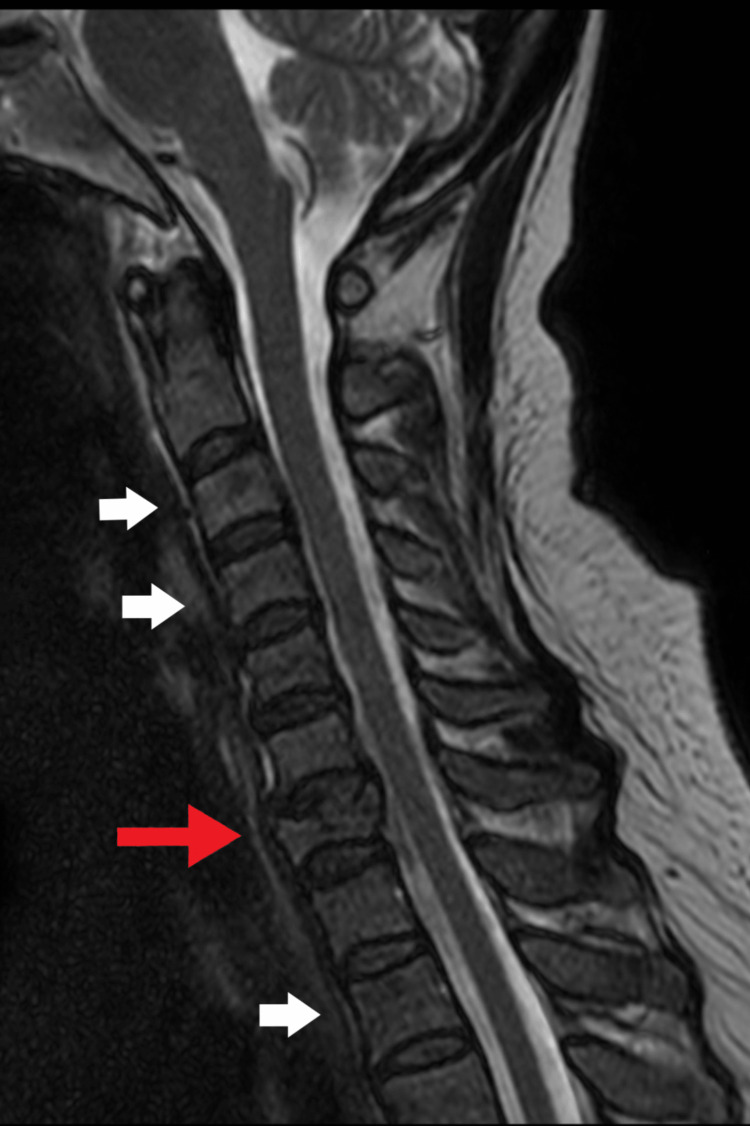
Sagittal view of the cervical MRI. Multiple patchy heterogeneous T1W hypointense T2W hyperintense marrow signals are visible at the cervical spine, including the upper thoracic spine and clivus. Partial collapse of the C7 vertebral body with mild retropulsion is noted. Possibility of bone metastasis/infiltrative marrow disease with pathological fracture of C7 was considered.

**Figure 2:**
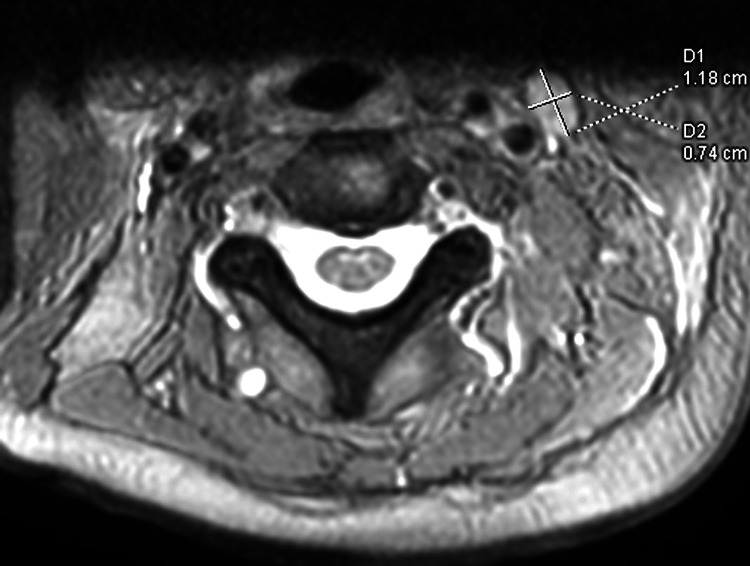
Coronal view of the cervical MRI. Few prominent left cervical lymph nodes (up 1.18 cm x 0.74 cm) are identified, which can represent metastatic or reactive node.

Magnetic resonance imaging of the cervical spine revealed T1-hypointense and T2-hyperintense bone marrow signal changes spanning several levels, with partial loss of vertebral body height and retropulsion of the C7 (Figure 1), prominent left cervical lymph nodes (up 1.18 cm x 0.74 cm) (Figure 2). The radiologist noted findings suggestive of an infiltrative bone marrow process with an associated pathological compression fracture at C7. Given the history of bilateral thyroid nodules, clinical presentation, and imaging showing an infiltrative bone marrow process with a pathological C7 compression fracture, the chiropractor diagnosed her with metastatic cancer involving the spine, possibly of thyroid origin. The patient was referred to an oncologist for further workup, including thyroid nodule and bone marrow biopsy, and imaging studies confirmed thyroid cancer. Radiotherapy and chemotherapy were initiated. Close follow-up and cancer management are ongoing. Traditional Chinese medicine treatments were discontinued because of the lack of benefits.

## Discussion

Spinal metastases are the most common tumors of the spine, comprising approximately 90% of the masses encountered on spinal imaging [7]. Spinal metastases typically spread through the vertebral bodies rather than the intervertebral discs [7]. Although primary cancers frequently metastasize to the spine, the least common, in ascending order, are thyroid cancer (2.5%), gastrointestinal malignancies (4.5%), renal cell carcinoma (5%), prostate adenocarcinoma (7.5%), pulmonary cancers (19%), and breast carcinoma (21%) [7]. Metastatic deposits tend to be least involved in the cervical spine, followed by the thoracic spine, and most affected in the lumbar region [7]. Therefore, our case of cervical spine metastasis from thyroid cancer is a rare incidence presenting at a chiropractic clinic.

The diagnosis of metastatic thyroid carcinoma was determined through a close clinical correlation of the patient's symptoms and medical history with the MRI results and tissue studies. The initial presentation with 2 months of severe progressive neck pain was suggestive of an underlying pathological process. A review of these systems revealed no overt signs of thyroid dysfunction or other malignancies. The patient's medical history of well-managed hypertension and hypercholesterolemia was unlikely to account for the current symptoms; however, bilateral thyroid nodules detected on prior ultrasonography prompted the suspicion of thyroid pathology.

Advanced imaging is essential for the early detection of abnormalities and diseases, allowing prompt intervention and treatment [8]. When substantial pain relief is not achieved with standard analgesics and conservative therapies, Hong Kong chiropractors often perform advanced imaging [9-14] to identify the defining features of spinal metastasis. The patient’s MRI confirmed an infiltrative bone marrow process and pathologic fracture. Fine-needle aspiration biopsy of the thyroid nodules and C7 vertebral body was performed by the oncologist to confirm the diagnosis. The cytological analysis was compatible with that of metastatic thyroid carcinoma. Subsequent histopathological evaluation of surgical biopsy specimens revealed features of papillary thyroid carcinoma. Multiple metastatic foci were detected on total body scintigraphy, and the patient was diagnosed with papillary thyroid carcinoma.

This case report demonstrates a rare initial presentation of metastatic thyroid cancer, primarily manifesting as neck pain. Chiropractors treat patients with cervicogenic pathologies, such as neck pain, vertigo, and headaches [15], and they are also exposed to spinal metastasis [16-17]. Early diagnosis of metastatic thyroid cancer is critical, as treatment options for advanced or progressive disease are limited, and the overall prognosis declines with a higher disease stage at diagnosis. As most chiropractors in Asia-Pacific reported avoiding manual therapy when a prospective treatment contraindication existed [18], a multidisciplinary approach is essential for accurate diagnosis and appropriate management without prolonged delays [7]. Chiropractors in Hong Kong frequently collaborate with medical physicians (18.82%), physiotherapists (9.41%), and acupuncturists (9.02%) to provide patients with comprehensive care [19], referral for advanced diagnostics, consultation with specialists, and close coordination of care, allowing for optimal evaluation and treatment of the patient’s complex condition. This case provides an important reminder that progressive or unresolved symptoms require serial reassessment to avoid missing occult diseases or alternative diagnoses, especially in high-risk patients. Metastatic thyroid carcinoma, although rare, remains an important differential diagnosis in such complex presentations.

## Conclusions

This case report highlights an unusual presentation of metastatic thyroid carcinoma manifesting primarily as progressive neck pain, emphasizing the importance of close follow-up and a high index of suspicion in patients with known risk factors such as thyroid disease. Despite the benign initial findings, the patient’s unremitting and severe symptoms prompted concern regarding occult malignancy, leading to the diagnosis of metastatic papillary thyroid cancer upon further evaluation and tissue sampling. Early detection is critical, as treatment options and prognosis decline in advanced stages. This case underscores the need to consider metastatic disease in patients with progressive symptoms and known primary cancers, to facilitate prompt diagnosis and management. It also provides an important reminder that undifferentiated neck pain warrants serial reassessment, especially in complex cases, to avoid missing alternative diagnoses or sinister pathologies. A multidisciplinary approach and open consideration of atypical disease manifestations are crucial when the initial workup is unclear. Close coordination of care between providers allowed for optimal diagnosis and treatment of this patient’s metastatic thyroid cancer. Overall, this case highlights the importance of clinician vigilance, follow-up of unresolved symptoms, and consideration of rare pathologies to minimize missed opportunities for early disease detection.
